# Social Media Fatigue and Depressive Symptoms Among Chinese University Students: Artificial Intelligence Anxiety as a Statistical Mediator and Subjective Socioeconomic Status as a Moderator

**DOI:** 10.3390/bs16101900

**Published:** 2026-10-14

**Authors:** Manlin Wu, Huimin Ma, Song Zhou, Lixian Yang

**Affiliations:** School of Psychology, Fujian Normal University, Fuzhou 350117, China; 117012024102@student.fjnu.edu.cn (M.W.); lixianyang@fjnu.edu.cn (L.Y.)

**Keywords:** social media fatigue, artificial intelligence anxiety, depressive symptoms, subjective socioeconomic status, moderated mediation

## Abstract

As social media and artificial intelligence (AI) become increasingly embedded in university students’ learning and daily lives, fatigue arising from established digital platforms and anxiety surrounding emerging AI may constitute interrelated forms of technology-related distress. However, how these experiences are jointly associated with depressive symptoms, and whether these associations vary by subjective socioeconomic status (SSES), remains insufficiently understood. Drawing on conservation of resources theory and research on status maintenance, this cross-sectional study examined AI anxiety as a statistical mediator of the association between social media fatigue and depressive symptoms and further tested whether SSES moderated the association between AI anxiety and depressive symptoms. Survey data from 1108 Chinese university students were analyzed using regression models with heteroscedasticity-consistent type 3 (HC3) robust standard errors and percentile-bootstrap confidence intervals. Social media fatigue was positively associated with AI anxiety, which in turn was positively associated with depressive symptoms, yielding a significant statistical indirect association. As SSES increased, the association between AI anxiety and depressive symptoms became stronger, and the corresponding conditional indirect association also increased. Overall, these findings suggest that understanding university students’ digital mental health requires attention to both distress arising across different technological environments and the subjective social positions from which these experiences are evaluated, offering a more context-sensitive perspective on how established and emerging forms of digital stress may be jointly associated with psychological well-being.

## 1. Introduction

Depressive symptoms, including core experiences such as low mood and diminished interest or pleasure, represent a prominent mental-health concern among university students, who must navigate academic demands, emerging adult roles, and an increasingly digitalized social environment (W. Li et al., 2022; Mason et al., 2025). Social media has become deeply embedded in this environment, yet its implications for mental health cannot be understood simply in terms of how often or how long students use digital platforms. Meta-analytic and review evidence on social media use and well-being, drawn from general and adolescent samples, indicates modest and heterogeneous associations, with substantial variation across patterns of use, user characteristics, and the experiences generated by platform engagement (Godard & Holtzman, 2024; Valkenburg et al., 2022). This has shifted attention from digital exposure itself toward the psychological demands associated with sustained connectivity, information overload, and continuous social interaction (Lee et al., 2016; Ou et al., 2023).

Social media fatigue is particularly relevant to this shift. Rather than merely indexing the volume of social media use, it captures the exhaustion and diminished psychological capacity that can accompany sustained engagement with social networking environments (Lee et al., 2016). Research in adolescent samples has linked social media fatigue with anxiety and depressive symptoms (Dhir et al., 2018), while evidence from Chinese university students similarly links social media fatigue with higher overall scores on the Depression Anxiety Stress Scales (DASS-21; Fan, 2022). These findings suggest that the mental-health relevance of social media may lie partly in the strain users experience while managing digital demands. Yet university students are no longer navigating social media in isolation. Their digital environment is being reshaped by the rapid diffusion of artificial intelligence (AI), particularly generative AI, into learning, assessment, information seeking, and anticipated employment (Eloundou et al., 2024; Kasneci et al., 2023; Y.-M. Wang et al., 2024).

The expansion of AI introduces a different form of technological uncertainty. Students are increasingly expected not only to use AI-enabled systems but also to anticipate how such systems may alter the skills required for study and work. Developments in generative AI have intensified concerns regarding occupational change and task automation (Eloundou et al., 2024), while research in educational settings shows that students’ engagement with AI is closely tied to their perceptions of its demands and consequences (Y.-M. Wang et al., 2024). Against this background, artificial intelligence anxiety has emerged as a technology-specific construct encompassing concerns about learning to use AI, potential job replacement, limited understanding of AI systems and their societal consequences, and the configuration or increasingly autonomous characteristics of such systems (Y.-Y. Wang & Wang, 2022). Unlike general technology anxiety or technostress, which concern apprehension toward technology more broadly and the wider strain generated by information-technology demands (Tarafdar et al., 2019), AI anxiety is directed specifically at the demands and threats associated with AI.

These strands of research raise an important but largely unanswered question. Social media fatigue and AI anxiety have typically been examined as responses to different aspects of digital life, despite being experienced within an increasingly integrated technological environment. Existing studies provide evidence for adjacent parts of a possible association pattern. Social media fatigue has been associated with psychological distress in both adolescent and university samples (Dhir et al., 2018; Fan, 2022). Among mainland Chinese users, AI-related information overload has been associated with greater AI anxiety (Qiu et al., 2025), while symptom-level research among Chinese university students has linked AI anxiety with depressive symptoms (Jin et al., 2026). What remains unclear is whether strain associated with an established digital environment is systematically related to how students respond to emerging AI-related demands, and whether these associations, when considered together, are related to depressive symptoms. A further open question is whether these associations are uniform across students, given that the psychological meaning of AI-related uncertainty may depend on the social position from which it is evaluated.

Drawing on conservation of resources theory (Hobfoll, 1989; Hobfoll et al., 2018) and research on status maintenance, with social comparison theory informing the relational conception of subjective socioeconomic status (Festinger, 1954), the present study examines whether AI anxiety statistically accounts for part of the association between social media fatigue and depressive symptoms and whether the association between AI anxiety and depressive symptoms varies with subjective socioeconomic status (SSES), understood here as perceived relative social standing.

### 1.1. Social Media Fatigue, Artificial Intelligence Anxiety, and Depressive Symptoms

Social media fatigue refers to a state of exhaustion arising from sustained engagement with social networking platforms and the demands embedded within them (Lee et al., 2016). Information overload, communication overload, constant connectivity, and platform-related demands have been identified as important antecedents of this form of fatigue (Lee et al., 2016; Ou et al., 2023; Sheng et al., 2023). Social media fatigue therefore captures more than heavy use: it reflects the extent to which engagement with digital platforms is experienced as taxing.

Conservation of resources theory provides a basis for explaining why such strain may be associated with anxiety about a different technological domain. The theory proposes that individuals strive to preserve valued resources and become increasingly sensitive to additional demands when those resources are threatened or depleted (Hobfoll, 1989; Hobfoll et al., 2018). Applied to the present context, students who experience social media engagement as exhausting may perceive additional technological demands as more difficult to manage when attentional or self-regulatory resources already feel strained. AI is especially relevant because adapting to it can involve learning unfamiliar systems, maintaining technological competence, interpreting opaque automated processes, and anticipating changes in future employment (Y.-Y. Wang & Wang, 2022).

Prior research has documented the antecedents and psychological correlates of social media fatigue (Lee et al., 2016; Dhir et al., 2018; Ou et al., 2023), while separate evidence indicates that AI-related information overload is associated with greater AI anxiety (Qiu et al., 2025). Direct evidence connecting social media fatigue with AI anxiety, however, remains limited. Extending conservation of resources theory to this emerging digital context, we therefore expect students reporting greater social media-related strain also to report greater anxiety about the additional demands represented by AI.

**H1.**  *Social media fatigue is positively associated with artificial intelligence anxiety.*

Artificial intelligence anxiety may, in turn, be relevant to depressive symptoms because it concerns domains central to students’ developing academic and occupational futures. The construct incorporates concerns about one’s ability to learn and adapt to AI as well as the possibility that intelligent systems will alter future employment opportunities (Y.-Y. Wang & Wang, 2022). Such concerns extend beyond immediate difficulty with technology and implicate broader evaluations of competence, security, and future prospects.

Anxiety and depressive symptoms are closely related but distinguishable forms of psychological distress. The tripartite model identifies substantial shared negative affect between anxiety and depression (Clark & Watson, 1991), and longitudinal evidence indicates that anxiety symptoms can prospectively precede increases in depressive symptoms, although reciprocal relationships also occur (Jacobson & Newman, 2017). More specifically, symptom-level research among Chinese university students has reported associations between artificial intelligence anxiety and depressive symptoms (Jin et al., 2026). Together, this evidence provides a basis for expecting stronger AI anxiety to be associated with greater depressive symptomatology.

**H2.**  *Artificial intelligence anxiety is positively associated with depressive symptoms.*

Integrating these arguments yields a theoretically ordered pattern in which social media fatigue is associated with AI anxiety and AI anxiety, in turn, is associated with depressive symptoms. The two forms of technology-related distress occupy different positions within this account: social media fatigue reflects strain arising from current digital engagement, whereas AI anxiety reflects apprehension about emerging technological demands. Examining AI anxiety as a statistical mediator therefore allows us to test whether the association between social media fatigue and depressive symptoms contains an indirect component consistent with this ordering. Because the focal constructs were measured concurrently, this ordering is theoretically motivated rather than temporally established; the indirect association therefore represents a statistical pattern rather than evidence of a causal mediation process. Reciprocal associations and shared negative affect remain plausible alternative accounts.

**H3.**  *Social media fatigue is indirectly and positively associated with depressive symptoms through artificial intelligence anxiety.*

### 1.2. The Moderating Role of Subjective Socioeconomic Status

The psychological significance of AI-related concerns may also depend on the social position from which students evaluate them. SSES captures individuals’ perceptions of their relative socioeconomic standing and therefore differs from purely objective indicators such as income or parental education (Antonoplis, 2023; Galvan et al., 2023). From a social comparison perspective, such perceptions are inherently relational: individuals evaluate their circumstances relative to salient social and economic reference groups (Festinger, 1954). SSES may therefore shape the standards and anticipated positions against which technological change is evaluated while also being associated with differences in perceived security and coping capacity.

Existing research gives rise to two competing expectations. A resource-buffering account suggests that higher socioeconomic standing may attenuate psychological responses to technological threat. Higher perceived standing is associated with more favorable economic circumstances and psychosocial profiles, whereas socioeconomic disadvantage and relative deprivation are consistently associated with poorer health and well-being (Cundiff et al., 2013; Dougall et al., 2024; Galvan et al., 2023; Kuo et al., 2024). From this perspective, students who perceive themselves as relatively advantaged may feel better positioned to accommodate uncertainty surrounding AI.

Social position, however, can also shape what is perceived to be at stake when future opportunities become uncertain. Research on status maintenance indicates that individuals are particularly sensitive to the prospect of losing an advantageous position. Pettit et al. (2010) found that avoiding status loss can carry greater motivational significance than obtaining an equivalent status gain, while Scheepers et al. (2009) showed that instability of a high-status position can elicit a threat response among members of relatively advantaged groups. Related research on downward mobility indicates that anticipated or experienced loss of socioeconomic advantage can generate collective angst among comparatively advantaged individuals (Jetten et al., 2021). Thus, socioeconomic advantage need not function exclusively as a psychological buffer; when valued future positions are perceived to be at risk, potential loss may acquire greater psychological salience.

The two accounts offer different perspectives on the role of perceived socioeconomic standing. A resource-buffering interpretation emphasizes resources that may help individuals accommodate technological change, such as financial security, social support, or AI literacy. SSES, however, captures perceived relative standing rather than these specific resources. This distinction is relevant to the associations examined in the present study. Social media fatigue reflects strain arising from existing digital engagement, and its association with AI anxiety may be understood in terms of the additional demands posed by emerging technologies. By contrast, the association between AI anxiety and depressive symptoms may also depend on how concerns about technological competence and future employment are evaluated in relation to one’s anticipated social position (Y.-Y. Wang & Wang, 2022). Perceived relative standing may therefore be particularly relevant to the psychological significance of AI anxiety.

For students reporting higher SSES, AI-related concerns may carry greater implications for maintaining anticipated academic and occupational trajectories. This reasoning does not imply that these students necessarily experience greater AI anxiety or more depressive symptoms. Rather, it suggests that, at a given level of AI anxiety, such concerns may be more strongly associated with depressive symptoms among students who perceive themselves as relatively advantaged. Although anticipated status loss and the personal significance of AI-related change were not directly measured, this interpretation provides a theoretical basis for the expected interaction. On this basis, we propose:

**H4.**  *The positive association between artificial intelligence anxiety and depressive symptoms is stronger at higher levels of subjective socioeconomic status.*

If the association between AI anxiety and depressive symptoms varies across levels of SSES, then the magnitude of the statistical indirect association linking social media fatigue with depressive symptoms through AI anxiety should vary accordingly. This conditional indirect association follows from the theoretically specified combination of the social media fatigue–AI anxiety association and the SSES-contingent AI anxiety–depressive symptoms association. As with H3, it represents a model-based statistical expectation rather than evidence of a temporally established moderated mediation process.

**H5.**  *The indirect association between social media fatigue and depressive symptoms through artificial intelligence anxiety is stronger at higher levels of subjective socioeconomic status.*

**Figure 1:**
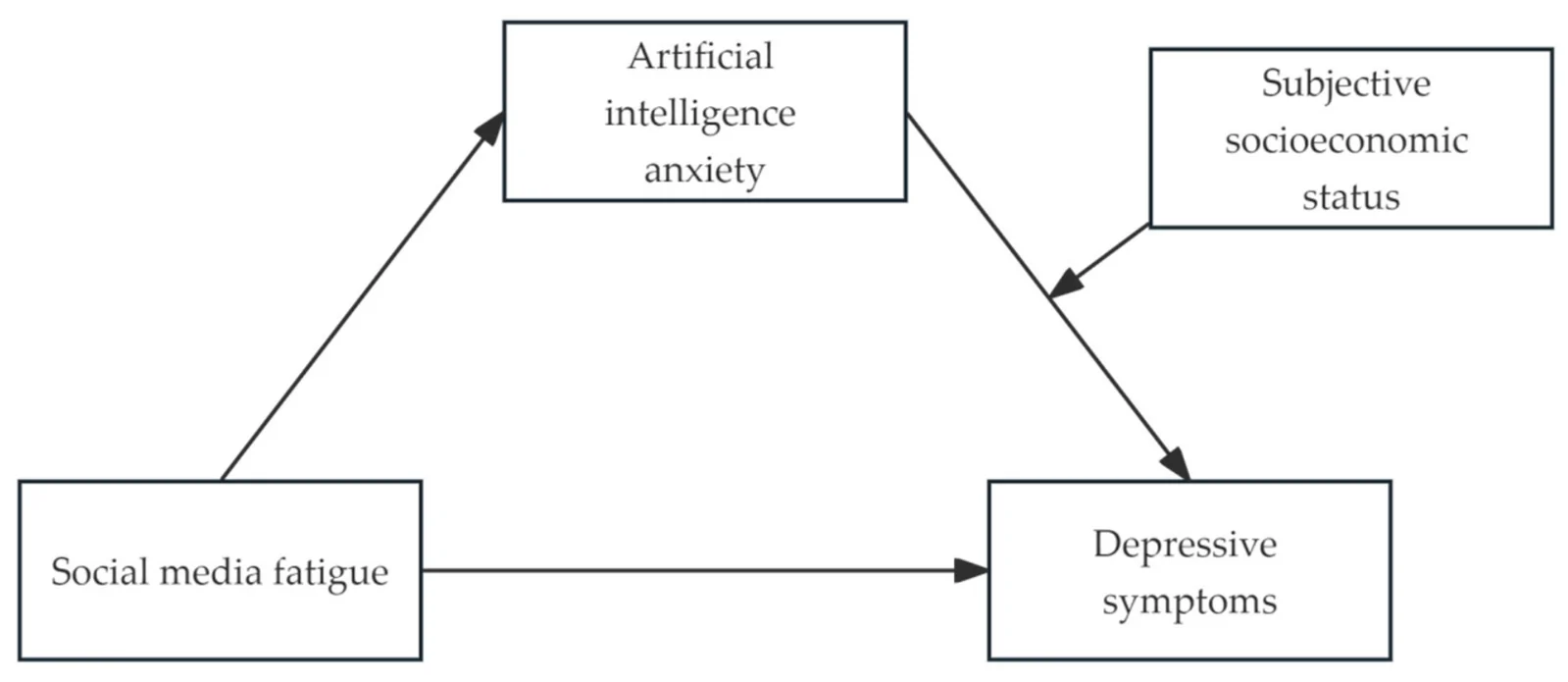
Conceptual model of the hypothesized moderated-mediation analysis. Artificial intelligence anxiety is modeled as a statistical mediator of the association between social media fatigue and depressive symptoms, and subjective socioeconomic status moderates the artificial-intelligence-anxiety–depressive-symptoms association. The lower horizontal path denotes the direct association between social media fatigue and depressive symptoms.

Taken together, the proposed model examines whether strain arising from established digital engagement and anxiety about an emerging technology are statistically connected and whether their association with depressive symptoms varies with perceived social position. The hypothesized model is presented in Figure 1.

## 2. Materials and Methods

### 2.1. Participants and Procedure

This cross-sectional online survey used convenience sampling to recruit first- and second-year university students aged 18 years or older from one university in Fujian Province, China. Recruitment notices were distributed through class WeChat and QQ groups and included a brief description of the study, eligibility requirements, the voluntary nature of participation, the estimated completion time, a link or QR code to the questionnaire, and compensation information. Data were collected through Wenjuanxing (https://www.wjx.cn/), an online survey platform, between 19 May and 31 October 2025. Each participant received RMB 1 after submitting the questionnaire.

A total of 1238 questionnaires were submitted. Before analysis, the questionnaires were screened sequentially for completeness, performance on instructed-response attention checks, invariant responding, and duplicate submissions. Each excluded record was counted only under the first criterion it met; thus, the reported exclusion categories were mutually exclusive. Specifically, one incomplete questionnaire was excluded, 52 questionnaires were excluded because they failed one instructed-response attention check, 75 were excluded because all items within at least one core multi-item scale received the same response, and two were removed because their responses were exact duplicates of another submission. In total, 130 questionnaires were excluded, leaving a final analytical sample of 1108 participants, corresponding to 89.50% of the submitted questionnaires.

The final sample included 690 men (62.27%) and 418 women (37.73%), aged 18–26 years (*M* = 19.57, *SD* = 1.45). All participants provided written informed consent before completing the survey and received remuneration after participation. The study was approved by the Ethics Committee of Fujian Normal University (protocol code 20210310 and date of approval 10 March 2021) and was conducted in accordance with the Declaration of Helsinki and relevant institutional ethical requirements.

### 2.2. Measures

#### 2.2.1. Social Media Fatigue

Social media fatigue was assessed using the five-item Chinese version of the Social Networking Service Fatigue Scale originally developed by Lee et al. (2016) and subsequently translated and used in a Chinese sample by K. Li et al. (2022). All items explicitly attributed the reported experience to social media use and assessed difficulty relaxing, fatigue or depletion after use, difficulty concentrating during leisure time, and insufficient energy for other activities. Responses were recorded on a five-point Likert scale ranging from 1 (strongly disagree) to 5 (strongly agree) and averaged, with higher scores indicating greater social media fatigue. Cronbach’s α was 0.944 in the present sample. The exact Chinese items, English translations, and supplementary measurement analyses are provided in Table S8.

#### 2.2.2. Artificial Intelligence Anxiety

Artificial intelligence anxiety was measured using the 21-item Artificial Intelligence Anxiety Scale developed by Y.-Y. Wang and Wang (2022). The scale comprises four related dimensions: learning anxiety (eight items), job-replacement anxiety (six items), sociotechnical-blindness anxiety (four items), and AI-configuration anxiety (three items). Items were rated on a five-point scale ranging from 1 (strongly disagree) to 5 (strongly agree). Because the primary hypotheses concerned AI anxiety as a broad construct rather than any single dimension, the mean of all 21 items was used in the primary analyses. Higher scores indicate greater AI anxiety. Cronbach’s α was 0.975 for the total score and ranged from 0.923 to 0.971 across the four dimensions. The multidimensional structure of the scale, higher-order reliability, and dimension-specific analyses are reported in Supplementary Section S2 and Tables S2 and S3.

#### 2.2.3. Depressive Symptoms

Depressive symptoms were assessed using the two-item Patient Health Questionnaire (PHQ-2; Kroenke et al., 2003). Participants reported how often during the preceding two weeks they had experienced depressed mood and diminished interest or pleasure. Each item was rated from 0 (not at all) to 3 (nearly every day), and the two items were summed to produce a score ranging from 0 to 6. Higher scores therefore represent greater frequency of these recent depressive symptoms. The PHQ-2 score was analyzed as a continuous measure of recent symptom frequency and was not interpreted as a diagnosis of depressive disorder. Cronbach’s α was 0.816 in the present sample.

#### 2.2.4. Subjective Socioeconomic Status

Subjective socioeconomic status (SSES) was assessed using two 10-rung ladder items adapted from the Chinese version of the Subjective Socioeconomic Status Scale (Chen et al., 2014). Following the China-specific reference-group modification described by Pan et al. (2022), one item assessed participants’ perceived family economic position relative to the socioeconomic development of their province, and the other assessed their perceived personal socioeconomic position relative to people around them. The original two-item Chinese scale has also been used in research with Chinese university students (Liu et al., 2022). Both items were rated on 10-point scales and averaged, with higher scores indicating higher perceived socioeconomic standing. Although the two items use different reference groups, both assess perceived relative position rather than objective income, wealth, or access to material resources. Cronbach’s α was 0.925 in the present sample.

### 2.3. Data Analysis

Analyses were conducted in R (v4.4.1) (R Core Team, 2024) and Mplus (v8.3) (Muthén & Muthén, 1998–2017). R was used for descriptive statistics, regression models, robust standard errors, bootstrap estimation, model diagnostics, and sensitivity analyses; confirmatory factor analyses (CFAs) were conducted in Mplus. Descriptive statistics, Pearson correlations, and internal-consistency estimates were calculated for the focal variables. Discriminant validity was examined using item-level heterotrait–monotrait ratios of correlations (HTMT; Henseler et al., 2015). Because all focal variables were collected by self-report in the same survey, we also compared a single-factor CFA with a theory-based seven-factor model comprising social media fatigue, the four AI-anxiety dimensions, depressive symptoms, and SSES. This comparison was used to assess whether a single undifferentiated factor could adequately reproduce the item covariance; it was not treated as evidence that common-method variance was absent. CFA fit was evaluated using the chi-square test (χ^2^), comparative fit index (CFI), Tucker–Lewis index (TLI), root mean square error of approximation (RMSEA), and standardized root mean square residual (SRMR).

The hypothesized conditional-process model corresponded to the statistical configuration represented by Hayes’s PROCESS Model 14. Instead, the mediator and outcome equations were estimated directly in R. Let *X* denote social media fatigue, *M* artificial intelligence anxiety, *W* subjective socioeconomic status, *Y* depressive-symptom frequency, and *C_k_* the covariates age and gender. The models were specified as

*M* = *a*_0_ + *a*_1_*X* + Σ*γ_k_C_k_* + *e_M_*

and

*Y* = *b*_0_ + *c*′*X* + *b*_1_*M* + *b*_2_*W* + *b*_3_(*M* × *W*) + Σ*δ_k_C_k_* + *e_Y_*.

Ordinary least squares regression was used with heteroscedasticity-consistent type 3 HC3 standard errors (J. S. Long & Ervin, 2000). Age and gender were included as basic demographic covariates in both equations. All continuous variables were standardized using the means and standard deviations of the final analytic sample before model estimation; the interaction term was calculated as the product of standardized AI anxiety and standardized SSES. Gender retained its original coding (1 = male, 2 = female). Coefficients for continuous predictors are therefore standardized coefficients, whereas the gender coefficient represents an adjusted group contrast in outcome standard-deviation units.

The conditional indirect association at a given value of SSES was calculated as

*ω*(*W*) = *a*_1_(*b*_1_ + *b*_3_*W*),

and the index of moderated mediation as *a*_1_*b*_3_ (Hayes, 2015).

Indirect associations and the index of moderated mediation were evaluated using 5000 percentile-bootstrap resamples with 95% confidence intervals. Both regression equations were re-estimated in each resample. Simple slopes of the AI-anxiety–depressive-symptom association were examined at −1 *SD*, the mean, and +1 *SD* of SSES, and the Johnson–Neyman procedure was used to identify the region of statistical significance for the continuous moderator (Hayes, 2013).

Regression diagnostics included variance inflation factors, heteroscedasticity assessment, studentized residuals, leverage, and Cook’s distance. Detailed diagnostic results are reported in the Supplementary Materials. Sensitivity analyses examined the four AI-anxiety dimensions, the two SSES items, models without age and gender, gender-specific moderation, and an alternative Poisson specification assessing the robustness of the results to treating the PHQ-2 sum score as a continuous outcome.

## 3. Results

### 3.1. Preliminary Analyses

Descriptive statistics and Pearson correlations are reported in Table 1. Across the focal variables, skewness ranged from 0.220 to 0.702 and excess kurtosis from 0.683 to 2.469. The PHQ-2 sum score ranged from 0 to 6 (*M* = 1.70, *SD* = 1.38). A total of 295 participants (26.6%) scored 0, the modal score was 2 (*n* = 482, 43.5%), and 205 participants (18.5%) scored 3 or higher. The complete observed distribution is reported in Table S9. Social media fatigue was positively correlated with artificial intelligence anxiety (*r* = 0.662, *p* < 0.001) and depressive symptoms (*r* = 0.284, *p* < 0.001). Artificial intelligence anxiety was also positively correlated with depressive symptoms (*r* = 0.327, *p* < 0.001). Subjective socioeconomic status was weakly correlated with social media fatigue (*r* = 0.133, *p* < 0.001) and artificial intelligence anxiety (*r* = 0.137, *p* < 0.001) but was not correlated with depressive symptoms (*r* = 0.027, *p* = 0.373). HTMT ratios ranged from 0.035 to 0.689 and were all below 0.85 (Henseler et al., 2015), supporting the empirical distinction among the four focal constructs (Table S1).

The single-factor CFA showed poor fit to the data, χ^2^(405) = 6077.520, *p* < 0.001, CFI = 0.598, TLI = 0.568, RMSEA = 0.112, 90% CI [0.110, 0.115], and SRMR = 0.104. In contrast, the theory-based seven-factor model showed an acceptable fit, χ^2^(384) = 1525.340, *p* < 0.001, CFI = 0.919, TLI = 0.908, RMSEA = 0.052, 90% CI [0.049, 0.055], and SRMR = 0.057. This comparison indicates that a single undifferentiated factor did not adequately reproduce the observed item covariance; it does not rule out shared reporting tendencies or other sources of common-method variance.

For the AI-anxiety measure, the correlated four-factor model fit somewhat better than the second-order model (CFI = 0.907 vs. 0.895; TLI = 0.894 vs. 0.880; RMSEA = 0.062 vs. 0.065; SRMR = 0.073 vs. 0.088). At the same time, omega hierarchical was 0.837, indicating substantial reliable variance shared across the four dimensions. Accordingly, the total score was treated as a broad composite of AI anxiety rather than as evidence of a strictly unidimensional construct. Full measurement-model results are reported in Table S2. The two SSES items were strongly correlated (*r* = 0.86). Analyses using the two items separately are reported in Table S10.

Before hypothesis testing, the outcome model was assessed for multicollinearity. All variance inflation factor (VIF) values were below 1.82, indicating no serious multicollinearity among the predictors.

### 3.2. Hypothesis Testing

Results of the simple mediation model are reported in Table 2. We first tested whether artificial intelligence anxiety statistically mediated the association between social media fatigue and depressive symptoms. Controlling for age and gender, social media fatigue was positively associated with artificial intelligence anxiety (β = 0.659, *SE* = 0.037, *p* < 0.001), and artificial intelligence anxiety was positively associated with depressive symptoms (β = 0.248, *SE* = 0.043, *p* < 0.001). The total association between social media fatigue and depressive symptoms was significant (β = 0.283, *SE* = 0.038, *p* < 0.001). After artificial intelligence anxiety was included in the model, the direct association remained significant (β = 0.120, *SE* = 0.041, *p* = 0.004). Bootstrap analysis indicated a significant standardized indirect association through artificial intelligence anxiety (β = 0.163, 95% CI [0.109, 0.223]). These findings suggest partial statistical mediation. These patterns were consistent with H1, H2, and H3.

**Figure 2:**
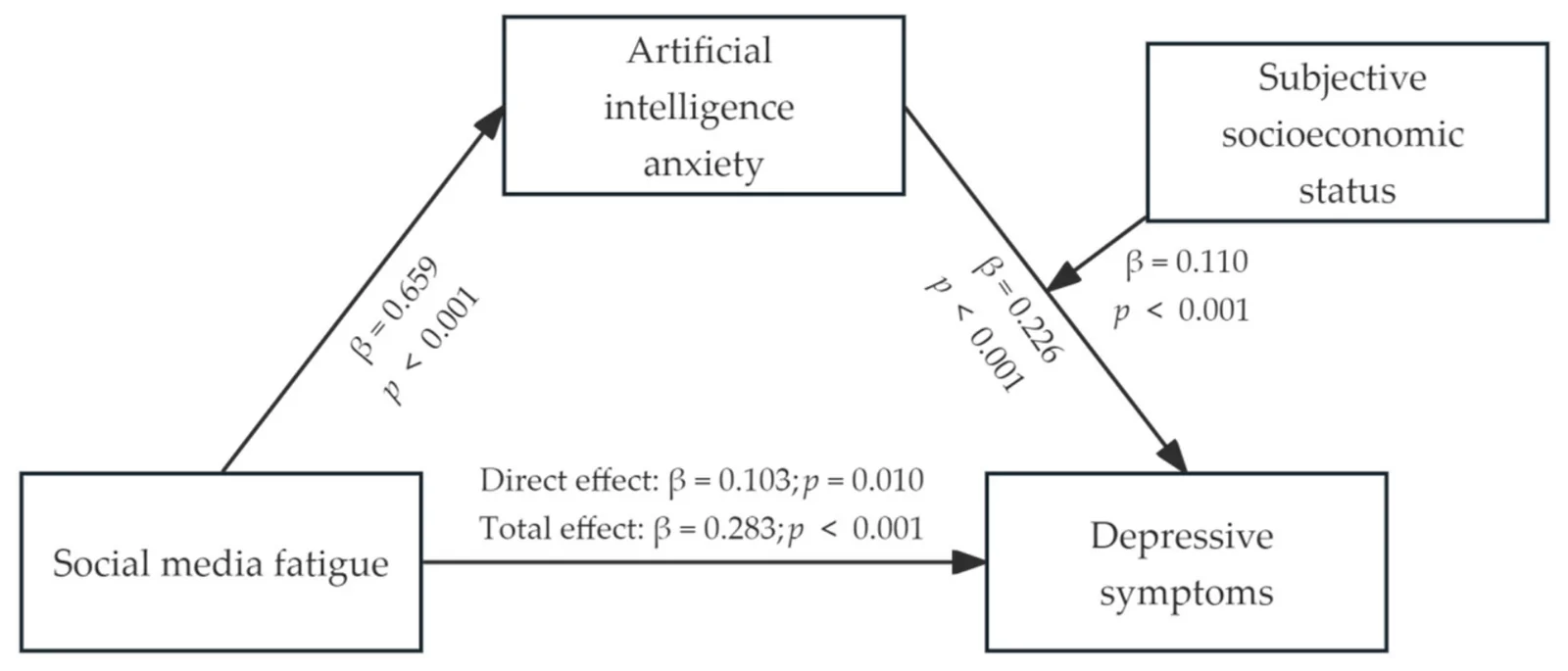
Standardized path coefficients for the moderated-mediation model. The coefficient beside the moderator arrow represents the artificial intelligence anxiety × subjective socioeconomic status interaction. The artificial-intelligence-anxiety–depressive-symptoms coefficient is evaluated at the mean of standardized subjective socioeconomic status. Age, gender, and the moderator’s main association were estimated but are omitted for clarity.

Results of the moderated-mediation regression model are reported in Table 3. We next examined whether subjective socioeconomic status moderated the association between artificial intelligence anxiety and depressive symptoms. In the outcome equation, artificial intelligence anxiety was positively associated with depressive symptoms (β = 0.226, *SE* = 0.041, *p* < 0.001), whereas subjective socioeconomic status was not significantly associated with depressive symptoms (β = −0.057, *SE* = 0.031, *p* = 0.071). Importantly, the interaction between artificial intelligence anxiety and subjective socioeconomic status was significant (β = 0.110, *SE* = 0.031, *p* < 0.001). The standardized path coefficients are summarized in Figure 2.

**Figure 3:**
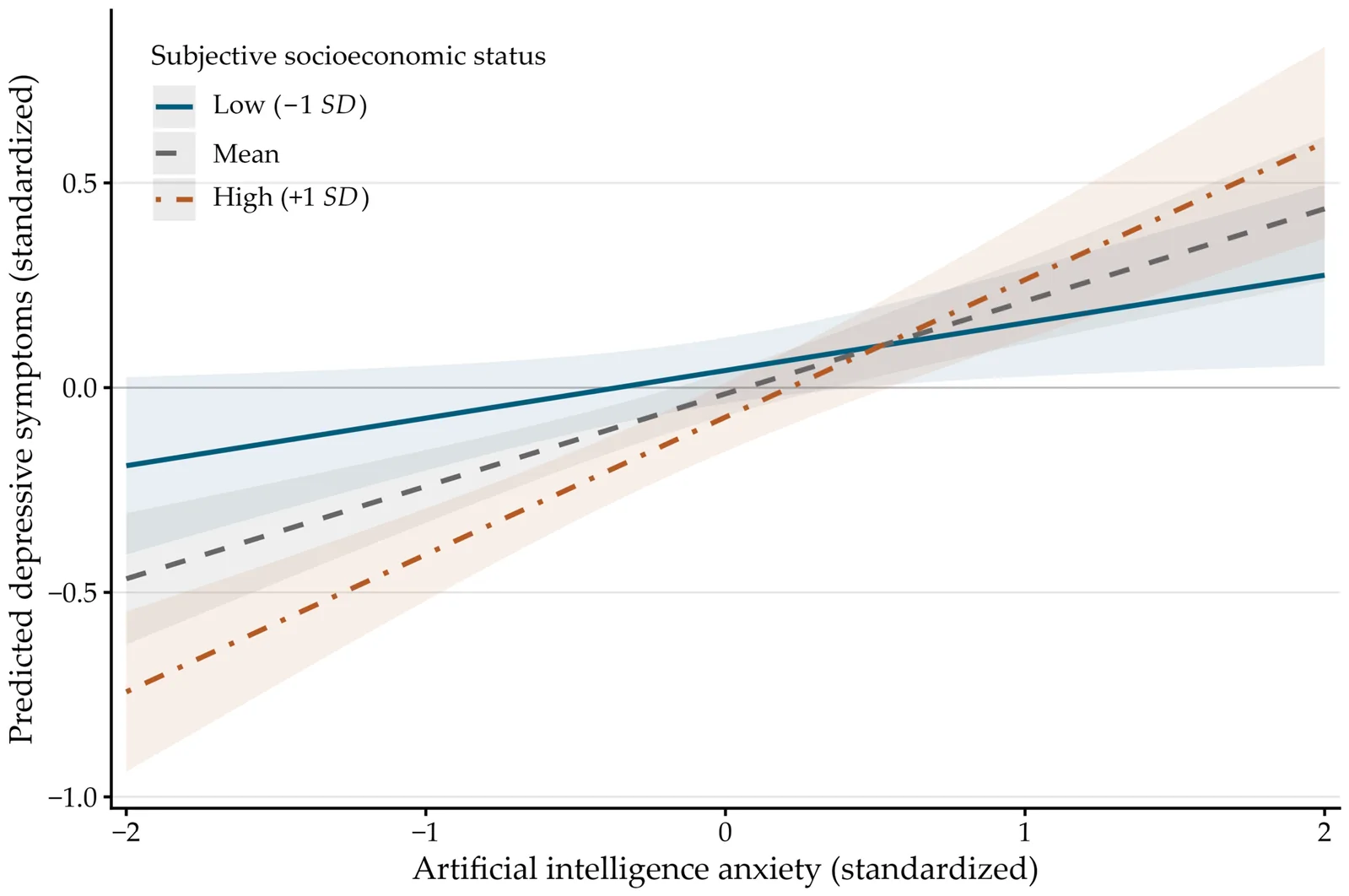
Simple slopes for the association between artificial intelligence anxiety and depressive symptoms at low (−1 standard deviation [*SD*]), mean, and high (+1 *SD*) levels of subjective socioeconomic status. Shaded areas represent 95% confidence intervals based on HC3 robust covariance estimates. Continuous variables were standardized; age and gender were controlled.

As shown in Figure 3, simple-slope analyses showed a graded pattern. The positive association between AI anxiety and depressive symptoms was weakest at low SSES (β = 0.116, *SE* = 0.052, 95% CI [0.015, 0.218], *p* = 0.025), stronger at the mean of SSES (β = 0.226, *SE* = 0.041, 95% CI [0.146, 0.306], *p* < 0.001), and strongest at high SSES (β = 0.335, *SE* = 0.051, 95% CI [0.236, 0.435], *p* < 0.001). The interaction accounted for an additional 2.2% of the variance in depressive symptoms (Δ*R*^2^ = 0.022; *f*^2^ = 0.025), indicating a small incremental contribution.

For interpretation in the original outcome metric, at the sample mean of SSES, the fitted model yielded an adjusted PHQ-2 contrast of 0.62 points, 95% CI [0.40, 0.84], between AI-anxiety values of −1 *SD* and +1 *SD*. This is a model-implied contrast rather than an observed difference between participant groups and should not be interpreted as clinically meaningful change. Corresponding contrasts at low and high levels of SSES are reported in Supplementary Table S6. This pattern was consistent with H4.

The Johnson–Neyman analysis identified a statistical significance threshold at −1.099 *SD* of SSES, corresponding to a raw SSES score of approximately 3.48 on the 1–10 scale. Because the observed two-item composite occurred in half-point increments, 120 participants (10.8%) with scores of 3.0 or lower fell below this threshold, whereas the conditional association was positive and statistically distinguishable from zero at observed scores of 3.5 or higher. However, this value represents a sample-specific region-of-significance boundary rather than a substantive socioeconomic cut-off.

The conditional indirect association between social media fatigue and depressive symptoms through AI anxiety also increased across levels of SSES. The standardized indirect association was 0.077 (95% CI [0.010, 0.144]) at low subjective socioeconomic status, 0.149 (95% CI [0.096, 0.206]) at the mean, and 0.221 (95% CI [0.156, 0.292]) at high subjective socioeconomic status (see Table 4). The difference between the high- and low-SSES conditional indirect associations was 0.144 (95% CI [0.068, 0.226]), and the index of moderated mediation was 0.072 (95% CI [0.034, 0.113]). These results were consistent with H5 and indicate that the model-based indirect association varied across levels of SSES.

The mediator equation explained 43.9% of the variance in AI anxiety, whereas the moderated outcome model explained 13.8% of the variance in depressive symptoms.

### 3.3. Sensitivity Analyses

Sensitivity analyses were conducted to examine whether the focal interaction depended on a particular measurement or model specification. The AI anxiety × SSES interaction was positive for each of the four AI-anxiety dimensions (βs = 0.104–0.123, all *p*s < 0.001; Table S3). The interaction was also positive when SSES was represented separately by perceived family standing or perceived personal standing (Table S10). The direction of the focal interaction was unchanged when age and gender were omitted and when the PHQ-2 sum score was analyzed using Poisson regression (Tables S4 and S5).

Given the unequal gender composition of the sample, exploratory gender analyses were also conducted. Men reported slightly higher AI anxiety than women (*M* = 3.08 vs. 2.95; Cohen’s *d* = 0.21). The AI anxiety × SSES interaction was positive within both gender groups, and the corresponding three-way AI anxiety × SSES × gender interaction was not statistically significant (*p* = 0.695). This result indicates that no gender difference in the focal interaction was detected in this sample. Full estimates are reported in the Supplementary Materials.

Taken together, the supplementary analyses indicate that the direction of the focal statistical interaction was not restricted to one AI-anxiety dimension, one SSES item, or the primary outcome-model specification.

## 4. Discussion

The present study examined how social media fatigue, artificial intelligence anxiety, and subjective socioeconomic status are jointly associated with depressive symptoms among university students. Three findings are central. First, greater social media fatigue was associated with greater AI anxiety. Second, AI anxiety was positively associated with depressive symptoms and statistically accounted for part of the association between social media fatigue and depressive symptoms. Third, the association between AI anxiety and depressive symptoms became stronger as subjective socioeconomic status increased, producing a corresponding increase in the conditional indirect association through AI anxiety. Together, these findings indicate that social media fatigue was positively associated with AI anxiety and that the association between AI anxiety and depressive symptoms varied across levels of subjective socioeconomic status.

### 4.1. Artificial Intelligence Anxiety in the Social Media Fatigue–Depressive Symptoms Association

The association between social media fatigue and AI anxiety extends existing research on technology-related distress in an important way. Previous studies have largely examined social media fatigue and AI anxiety within separate technological contexts: the former in relation to sustained social media engagement and the latter in relation to emerging AI-related demands. The present findings suggest that these experiences may nevertheless be connected. From a conservation of resources perspective, social media fatigue may indicate that sustained connectivity, information overload, and communication demands are experienced as taxing (Hobfoll, 1989; Hobfoll et al., 2018; Lee et al., 2016). When existing digital demands are experienced in this way, additional technological demands may also be appraised as more difficult to manage. Concerns about learning unfamiliar AI systems, maintaining technological competence, or adapting to changes in future employment may therefore become more salient among students who already experience their digital environment as demanding. The association reported by Qiu et al. (2025) between AI-related information overload and AI anxiety is consistent with this broader interpretation that demanding digital environments may be related to greater AI-specific apprehension.

This finding is theoretically relevant because social media fatigue and AI anxiety represent different forms of technology-related distress rather than interchangeable manifestations of the same construct. Social media fatigue is anchored in strain associated with current and repeated platform engagement, whereas AI anxiety is oriented toward the demands and possible consequences of an emerging technology. Their association therefore suggests that strain experienced in one part of the digital environment may be relevant to how students respond to demands arising in another. In this sense, the present findings extend the study of digital stress beyond isolated platforms or technologies toward a more integrated account of how established and emerging technological demands may intersect.

AI anxiety was also positively associated with depressive symptoms. This finding is consistent with previous evidence among Chinese university students linking AI anxiety with depressive symptomatology (Jin et al., 2026) and with broader evidence that anxiety and depressive symptoms are closely related forms of psychological distress (Clark & Watson, 1991; Jacobson & Newman, 2017). The content of AI anxiety may be particularly relevant during university because it concerns domains central to students’ developing academic and occupational futures. Questions about whether one can acquire adequate AI-related competence, remain competitive in changing educational environments, or preserve future employment opportunities may extend beyond immediate technological discomfort and become connected with broader evaluations of competence, security, and future prospects (Y.-Y. Wang & Wang, 2022).

Considered together, these findings are consistent with the theoretical distinction proposed in the Introduction between experienced digital strain and AI-specific anxiety. The indirect association involving AI anxiety indicates a statistical pattern in which strain associated with current digital engagement is related to stronger apprehension about emerging technological demands, which is also associated with greater depressive symptomatology. The remaining direct association between social media fatigue and depressive symptoms indicates that AI anxiety statistically accounts for only part of this relationship. Rather than demonstrating a single explanatory pathway, the results suggest that AI anxiety may be one relevant component within a broader pattern of associations between digital strain and student mental health.

### 4.2. Subjective Socioeconomic Status as a Boundary Condition

The moderating role of subjective socioeconomic status adds an important qualification to this pattern. AI anxiety was positively associated with depressive symptoms across most of the observed range of SSES, and this association became stronger as perceived socioeconomic standing increased. The interaction made only a small incremental contribution to explained variance (Δ*R*^2^ = 0.022; *f*^2^ = 0.025); SSES should therefore be viewed as a contextual qualifier rather than a dominant determinant of depressive symptoms. Nevertheless, the direction of the interaction warrants consideration because it runs counter to a straightforward resource-buffering expectation.

A resource-buffering account would suggest that the resources and psychosocial advantages commonly associated with higher perceived standing may make students less psychologically affected by AI-related concerns (Cundiff et al., 2013; Dougall et al., 2024; Galvan et al., 2023; Kuo et al., 2024). The observed pattern did not follow this expectation. One possible interpretation is provided by the status-maintenance account. SSES reflects perceived relative position rather than objective material resources, and AI anxiety includes concerns about competence and future employment that may be relevant to maintaining desired academic and occupational trajectories. From this perspective, AI-related concerns may carry greater self-evaluative significance among students who perceive themselves as occupying a relatively advantaged position.

This possibility is consistent with research showing that potential status loss can have psychological consequences for individuals who occupy or expect to occupy relatively advantaged positions. Pettit et al. (2010) found that avoiding status loss can carry greater motivational significance than achieving an equivalent status gain, while Scheepers et al. (2009) showed that instability in a high-status position can elicit threat responses among advantaged groups. Jetten et al. (2021) similarly demonstrated that anticipated or experienced downward mobility can generate collective angst among relatively advantaged individuals. Applied to the present context, students with higher perceived socioeconomic standing may attach greater importance to AI-related concerns when they perceive those concerns as relevant to maintaining competence, competitiveness, or future educational and occupational opportunities. This literature provides a plausible basis for the status-maintenance interpretation but does not establish it as the mechanism underlying the observed interaction.

The dimension-specific analyses further qualify this interpretation. The positive interaction across all four dimensions indicates that the composite finding was not confined to a single component of AI anxiety. However, the status-maintenance account is most directly relevant to learning anxiety and job-replacement anxiety because these dimensions concern adaptation, competence, and future occupational prospects. Its connection with sociotechnical-blindness anxiety and AI-configuration anxiety is less direct, as these dimensions also encompass concerns about the broader societal consequences, potential misuse, and characteristics of AI systems (Y.-Y. Wang & Wang, 2022). Thus, the consistency across dimensions supports the robustness of the interaction but does not specifically identify status maintenance as its explanation. Broader AI-related threat appraisal and other unmeasured processes remain possible explanations.

At the same time, the weaker association at lower SSES should not be interpreted as evidence that students with lower perceived standing are protected from AI-related distress. One possibility is that more immediate financial, academic, or employment-related pressures account for a larger share of depressive symptoms among these students, leaving AI-specific concerns relatively less predictive of variation in depressive symptoms. These competing pressures were not measured in the present study, so this interpretation remains provisional. The broader literature consistently links socioeconomic disadvantage with poorer mental health and well-being (Dougall et al., 2024), so the present interaction should not be read as reversing that general socioeconomic gradient. Rather, it concerns the relative strength of one specific association—between AI anxiety and depressive symptoms—across levels of perceived socioeconomic position.

The moderated indirect association followed the same statistical pattern. Social media fatigue was positively associated with AI anxiety, and the association between AI anxiety and depressive symptoms was stronger at higher levels of SSES; accordingly, the estimated indirect association involving AI anxiety also increased with perceived socioeconomic standing. This extends the moderation result from a single association to the broader conditional model, showing that the strength of the estimated indirect association varied across levels of SSES.

### 4.3. Theoretical and Practical Implications

The study contributes to research on digital well-being in two respects. First, it connects strain associated with an established digital environment with anxiety surrounding an emerging technology while preserving the conceptual distinction between the two. This suggests that technology-related distress may be better understood not only in relation to individual platforms or technologies but also in terms of how different digital demands coexist within students’ everyday environments.

Second, the findings indicate that perceived socioeconomic status may serve as a boundary condition in the association between technology-related anxiety and depressive symptoms. Rather than being uniformly weaker among students reporting greater perceived advantage, the association between AI anxiety and depressive symptoms was stronger at higher levels of SSES. This highlights the importance of considering perceived social position when examining the association between technology-related concerns and mental health outcomes.

The practical implications should be interpreted cautiously. The present findings do not demonstrate that reducing social media fatigue or AI anxiety would reduce depressive symptoms, nor do they establish that students should be assigned support according to a particular SSES threshold. They nevertheless identify possible directions for future intervention research. Universities could evaluate a layered, needs-responsive model. At a universal level, this could include clear guidance and transparent expectations regarding acceptable AI use (Chan, 2023), foundational education concerning system opacity, limitations, data practices, and uncertainty in automated outputs (Kasneci et al., 2023; D. Long & Magerko, 2020), and accessible mental-health support for technology-related stress. Where additional needs are identified, more flexible assessment options, targeted AI-skills assistance, technical or material support, and career counseling focused on technological change could be provided and evaluated. Support should be guided by directly assessed academic, financial, technical, and psychological needs rather than by the sample-specific Johnson–Neyman threshold. Because the effectiveness of these approaches was not examined in the present study, they require prospective or controlled evaluation.

### 4.4. Limitations and Future Directions

Several limitations qualify these conclusions. First, the cross-sectional design does not establish temporal ordering or causal mediation. Although the proposed ordering from social media fatigue to AI anxiety to depressive symptoms is theoretically motivated, the variables were measured concurrently. Depressive symptoms may heighten perceived social media fatigue or AI-related worry; the associations may be reciprocal, or all three may be influenced by other unmeasured factors. Cross-sectional indirect effects can differ from the longitudinal processes they are intended to represent (Maxwell & Cole, 2007). Longitudinal, intensive repeated-measures, and experimental designs are therefore needed to examine temporal sequencing and within-person change.

Second, all focal variables were assessed by self-report within the same survey. Common-method variance and shared negative affect may therefore contribute to the observed associations, particularly between social media fatigue and AI anxiety (Podsakoff et al., 2003). Although the measurement analyses supported their empirical distinction, the present design cannot determine how much of their association reflects substantive overlap versus shared response tendencies. Future studies could strengthen inference through temporal separation, behavioral measures of digital exposure, repeated assessments, or additional informants where appropriate.

Third, several theoretically relevant variables were not measured. The resource-depletion interpretation assumes differences in available coping resources; the threat-appraisal interpretation assumes differences in how AI-related concerns are evaluated; and the status-maintenance interpretation invokes achievement expectations, anticipated status loss, self-evaluative meaning, and concerns about future position. These processes remain theoretical explanations rather than mechanisms directly tested in the present study. Prior mental health difficulties, general anxiety, financial strain, objective socioeconomic indicators such as parental income and parental education, social media exposure, actual AI use frequency, performance-based AI literacy, coping resources, and institutional AI environments may also confound or modify the observed associations. Future studies should measure these constructs directly to distinguish among competing explanations and determine whether the SSES interaction remains after objective socioeconomic conditions are taken into account.

Fourth, the measurement and sampling strategy constrain generalizability. Although the dimension-specific analyses indicated that the moderation pattern was not confined to one component of AI anxiety, the composite score may obscure differences among the four dimensions. Participants were first- and second-year students recruited through compensated online convenience sampling from a single university in Fujian Province, and the sample was predominantly male. The exploratory analyses did not detect a gender difference in the AI anxiety × SSES interaction, but this does not establish equivalence across gender groups. The academic field was not recorded, so possible overrepresentation of technology-related disciplines could not be assessed. Future multi-institutional studies should use more gender-balanced samples and collect information on academic field, prior AI training, actual AI use, and institutional AI policies. Sampling sufficient numbers of majors and universities would also permit multilevel analyses of disciplinary and institutional contexts.

Fifth, depressive symptoms were measured using the PHQ-2, which assesses the recent frequency of depressed mood and diminished interest or pleasure rather than providing a comprehensive clinical assessment of depression (Kroenke et al., 2003). The findings should therefore be interpreted in terms of recent depressive-symptom frequency rather than depressive disorder or clinical change.

Finally, the outcome model explained 13.8% of the variance in depressive symptoms, indicating that most individual variation remained unexplained. The model should consequently be understood as identifying one conditional technology-related association pattern rather than providing a comprehensive account of student depressive symptoms.

## 5. Conclusions

This cross-sectional study identified a positive statistical indirect association between social media fatigue and the frequency of low mood and diminished interest or pleasure measured by the PHQ-2 through artificial intelligence anxiety. The positive association between artificial intelligence anxiety and PHQ-2 symptom frequency was stronger at higher levels of subjective socioeconomic status, resulting in larger model-based conditional indirect associations at higher SSES. These findings bring together strain associated with established digital engagement, anxiety surrounding emerging AI-related demands, and perceived socioeconomic position within a single conditional association model. They do not establish temporal or causal mediation, and the psychological processes proposed to account for the observed moderation were not directly measured. Longitudinal and experimental research is needed to establish temporal ordering and directly test the proposed mechanisms.

## Figures and Tables

**Table 1:** Descriptive statistics, internal consistency, and Pearson correlations.

Variable	*M*	*SD*	1	2	3	4
1. Social media fatigue	3.06	0.72	0.944			
2. Artificial intelligence anxiety	3.03	0.67	0.662 ***	0.975		
3. Depressive symptoms	1.70	1.38	0.284 ***	0.327 ***	0.816	
4. Subjective socioeconomic status	5.48	1.82	0.133 ***	0.137 ***	0.027	0.925

Note. *N* = 1108. M = mean; SD = standard deviation. Means and standard deviations are reported in the original response units. Cronbach’s α coefficients appear on the diagonal. *** *p* < 0.001.

**Table 2:** Statistical indirect-association analysis.

Association	β	HC3 *SE*	*t*	*p*	95% CI	*R* ^2^
Social media fatigue → Artificial intelligence anxiety	0.659	0.037	17.696	<0.001	[0.585, 0.732]	0.439
Artificial intelligence anxiety → Depressive symptoms	0.248	0.043	5.722	<0.001	[0.163, 0.333]	0.115
Total association: Social media fatigue → Depressive symptoms	0.283	0.038	7.441	<0.001	[0.209, 0.358]	0.081
Direct association: Social media fatigue → Depressive symptoms	0.120	0.041	2.899	0.004	[0.039, 0.202]	0.115
Indirect association through artificial intelligence anxiety	0.163	0.029 ^a^	—	—	[0.109, 0.223] ^a^	—

Note. *N* = 1108. Continuous variables were standardized before model estimation. Regression coefficients are standardized coefficients estimated with heteroscedasticity-consistent type 3 (HC3) robust standard errors. *SE* = standard error; CI = confidence interval; *R*^2^ = coefficient of determination. Superscript ^a^ identifies the bootstrap standard error and percentile-bootstrap confidence interval based on 5000 resamples. Age and gender were included as covariates.

**Table 3:** Moderated-mediation regression model predicting depressive symptoms.

Predictor	β	HC3 *SE*	*t*	*p*	95% CI
Social media fatigue	0.103	0.040	2.593	0.010	[0.025, 0.181]
Artificial intelligence anxiety	0.226	0.041	5.534	<0.001	[0.146, 0.306]
Subjective socioeconomic status	−0.057	0.031	−1.810	0.071	[−0.119, 0.005]
Artificial intelligence anxiety × Subjective socioeconomic status	0.110	0.031	3.525	<0.001	[0.049, 0.171]
Gender	0.016	0.055	0.296	0.768	[−0.092, 0.124]
Age	−0.002	0.029	−0.077	0.939	[−0.059, 0.055]

Note. *N* = 1108. Continuous variables were standardized before model estimation. Coefficients for continuous predictors were estimated as standardized coefficients with HC3 robust standard errors. Gender was coded 1 = male and 2 = female; its coefficient represents an adjusted group contrast in outcome standard-deviation units. Model *R*^2^ = 0.138; change in *R*^2^ attributable to the interaction = 0.022; *f*^2^ = 0.025.

**Table 4:** Conditional associations and the index of moderated mediation.

Effect	Level of Subjective Socioeconomic Status	Estimate	*SE*	*t*	*p*	95% CI
Simple slope	Low (−1 *SD*)	0.116	0.052	2.245	0.025	[0.015, 0.218]
Simple slope	Mean	0.226	0.041	5.534	<0.001	[0.146, 0.306]
Simple slope	High (+1 *SD*)	0.335	0.051	6.604	<0.001	[0.236, 0.435]
Conditional indirect association	Low (−1 *SD*)	0.077	0.034 ^a^	—	—	[0.010, 0.144] ^a^
Conditional indirect association	Mean	0.149	0.028 ^a^	—	—	[0.096, 0.206] ^a^
Conditional indirect association	High (+1 *SD*)	0.221	0.035 ^a^	—	—	[0.156, 0.292] ^a^
Conditional indirect association	High minus low	0.144	0.040 ^a^	—	—	[0.068, 0.226] ^a^
Moderated mediation	Index	0.072	0.020 ^a^	—	—	[0.034, 0.113] ^a^

Note. *N* = 1108. Simple-slope standard errors and confidence intervals are based on HC3 robust covariance estimates. Superscript ^a^ identifies bootstrap standard errors and percentile-bootstrap confidence intervals based on 5000 resamples. *SE* = standard error; CI = confidence interval.

## Data Availability

The datasets generated during the current study are available in the Harvard Dataverse repository at https://doi.org/10.7910/DVN/UBHQSQ.

## Supplementary Materials

The following supporting information can be downloaded at: https://www.mdpi.com/article/10.3390/bs16101900/s1, Section S1: Discriminant Validity; Table S1: Pairwise heterotrait–monotrait ratios of correlations; Section S2: Measurement Structure of Artificial Intelligence Anxiety; Table S2: Fit of alternative artificial intelligence anxiety measurement models; Section S3: Dimension-Specific Sensitivity Analyses; Table S3: Complete outcome equations for dimension-specific sensitivity analyses; Section S4: Models without Demographic Covariates; Table S4: Outcome equation without age and gender; Section S5: Distributional Sensitivity for the PHQ-2 Outcome; Table S5: Poisson sensitivity model for the PHQ-2 sum score; Section S6: Johnson–Neyman Analysis; Table S6: Conditional AIA associations and model-implied contrasts in original PHQ-2 units; Section S7: Software and Reproducibility; Section S8: Social Media Fatigue Items and Measurement Evidence; Table S8: Social media fatigue items and one-factor measurement model; Section S9: Distribution of PHQ-2 Sum Scores; Table S9: Distribution of PHQ-2 sum scores; Section S10: Subjective Socioeconomic Status Items and Item-Specific Analyses; Table S10: Exact SSES item wording and item-specific moderation analyses; Section S11: Regression Diagnostics and Additional Sensitivity Analyses; Table S11: Regression diagnostics and additional sensitivity analyses.
