# Supplementary material for: Social Media Fatigue and Depressive Symptoms Among Chinese University Students: Artificial Intelligence Anxiety as a Statistical Mediator and Subjective Socioeconomic Status as a Moderator
Source: Behav Sci (Basel). 2026 Oct 14;16(10):1900. doi: 10.3390/bs16101900 (PMC13701309; doi:10.3390/bs16101900)
Supplement: Supplementary file 1 [file behavsci-16-01900-s001.zip › behavsci-4593017-supplementary.pdf]

Supplementary Materials

S1. Discriminant Validity

S1.1. Supplementary Methods

To assess whether the four focal constructs in the regression analyses were empirically distinguishable, we calculated heterotrait–monotrait ratios of correlations (HTMT) for social media fatigue, artificial intelligence anxiety, depressive symptoms, and subjective socioeconomic status. Each ratio was derived from the absolute item-level Pearson correlations between a pair of constructs and the corresponding within-construct item correlations. Following Henseler et al. (2015), values below 0.85 were taken to indicate adequate discriminant validity.

S1.2. Supplementary Results

As shown in Table S1, the six HTMT ratios ranged from 0.035 to 0.689. The largest ratio was observed between social media fatigue and artificial intelligence anxiety (HTMT = 0.689), whereas all remaining ratios were 0.366 or lower. This pattern supports the empirical separation of the four constructs used in the regression analyses. Because HTMT does not establish the internal structure of a multidimensional measure, the measurement structure of artificial intelligence anxiety was examined separately in Section S2.

Table S1. Pairwise heterotrait–monotrait ratios of correlations.

Construct 1	Construct 2	HTMT
Social media fatigue	Artificial intelligence anxiety	0.689
Social media fatigue	Depressive symptoms	0.324
Social media fatigue	Subjective socioeconomic status	0.142
Artificial intelligence anxiety	Depressive symptoms	0.366
Artificial intelligence anxiety	Subjective socioeconomic status	0.144
Depressive symptoms	Subjective socioeconomic status	0.035

Note. HTMT = heterotrait–monotrait ratio of correlations. Ratios were calculated from absolute item-level Pearson correlations.

S2. Measurement Structure of Artificial Intelligence Anxiety

S2.1. Supplementary Methods

The primary analyses represented artificial intelligence anxiety (AIA) by the mean of its 21 items. Because the scale comprises four related dimensions—learning anxiety, job-replacement anxiety, sociotechnical-blindness anxiety, and AI-configuration anxiety—we examined whether the items also retained sufficient common variance to support a broad composite score. Two confirmatory factor models were estimated in Mplus (v8.3) with robust maximum likelihood (MLR). The first specified four correlated first-order factors; the second retained these factors and modeled them as indicators of a single second-order AIA factor. No residual covariances were added. Model fit was evaluated using chi-square, CFI, TLI, RMSEA with its 90% confidence interval, and SRMR.

To complement the factor analyses, omega reliability coefficients were estimated in R (v4.4.1) with psych (v2.5.3) using minimum-residual extraction and four group factors. Omega total estimates the reliability attributable to all common sources of variance in the composite, whereas omega hierarchical isolates the variance attributable to a general factor after the group factors are taken into account.

S2.2. Supplementary Results

As shown in Table S2, the correlated four-factor model fit the data better than the second-order model. In the second-order solution, the standardized higher-order loadings were 0.714 for learning anxiety, 0.979 for job-replacement anxiety, 0.967 for sociotechnical-blindness anxiety, and 0.829 for AI-configuration anxiety; all were statistically significant (*p*s < 0.001). Reliability was high at both the total-score level ($\omega_{total} = 0.987$) and the general-factor level ($\omega_H =$

0.837). Taken together, these results indicate substantial shared variance across the four dimensions while also showing that the dimensions are not interchangeable. The broad composite was therefore retained for the primary analysis, and the four dimensions were examined separately in Section S3.

Table S2. Fit of alternative artificial intelligence anxiety measurement models.

Model	χ^2	*df*	CFI	TLI	RMSEA (90% CI)	SRMR
Correlated four-factor	949.905	183	0.907	0.894	0.062 [0.058, 0.065]	0.073
Second-order AIA	1055.821	185	0.895	0.880	0.065 [0.061, 0.069]	0.088

Note. Both chi-square tests had $p < 0.001$. CFI = comparative fit index; TLI = Tucker–Lewis index; RMSEA = root mean square error of approximation; SRMR = standardized root mean square residual; MLR = robust maximum likelihood.

S3. Dimension-Specific Sensitivity Analyses

S3.1. Supplementary Methods

To determine whether the interaction observed for the overall AIA score was concentrated in a particular component of the scale, we re-estimated the moderated outcome equation four times, substituting one AIA dimension at a time. Item responses were reconstructed in their original 1–5 response units, averaged within each dimension, and standardized. Each model included social media fatigue, the selected AIA dimension, subjective socioeconomic status (SSES), the dimension × SSES product term, gender, and standardized age. HC3 covariance estimates were used for standard errors and confidence intervals. These models were treated as sensitivity analyses rather than as separate hypothesis tests.

S3.2. Supplementary Results

Table S3 presents the complete dimension-specific outcome models. The interaction between SSES and each AIA dimension was positive and statistically significant (βs = 0.104–0.123, *p*s < 0.001). The consistency of this pattern indicates that the interaction observed for the overall AIA score was not attributable exclusively to one dimension.

Table S3. Complete outcome equations for dimension-specific sensitivity analyses.

AIA dimension	Predictor	β	HC3 *SE*	*t*	*p*	95% CI	R^2
Learning anxiety	Intercept	−0.055	0.083	−0.666	0.506	[−0.218, 0.107]	0.137
	Social media fatigue	0.123	0.039	3.120	0.002	[0.046, 0.201]	
	AIA dimension	0.207	0.038	5.446	<0.001	[0.132, 0.281]	
	Subjective socioeconomic status	−0.062	0.032	−1.960	0.050	[−0.125, 0.000]	
	AIA dimension × SSES	0.123	0.033	3.773	<0.001	[0.059, 0.187]	
	Gender	0.028	0.055	0.501	0.616	[−0.080, 0.136]	
	Age	−0.005	0.029	−0.174	0.862	[−0.062, 0.052]	
Job-replacement anxiety	Intercept	−0.009	0.084	−0.109	0.913	[−0.174, 0.156]	0.127

AIA dimension	Predictor	β	HC3 *SE*	*t*	*p*	95% CI	R^2
	Social media fatigue	0.143	0.038	3.760	<0.001	[0.068, 0.217]	
	AIA dimension	0.192	0.037	5.196	<0.001	[0.119, 0.264]	
	Subjective socioeconomic status	−0.047	0.032	−1.467	0.143	[−0.110, 0.016]	
	AIA dimension × SSES	0.108	0.030	3.569	<0.001	[0.049, 0.168]	
	Gender	−0.002	0.056	−0.041	0.967	[−0.111, 0.107]	
	Age	−0.001	0.029	−0.035	0.972	[−0.058, 0.056]	
Sociotechnical-blindness anxiety	Intercept	−0.005	0.085	−0.057	0.954	[−0.171, 0.161]	0.114
	Social media fatigue	0.180	0.037	4.865	<0.001	[0.107, 0.252]	
	AIA dimension	0.132	0.036	3.666	<0.001	[0.061, 0.203]	
	Subjective socioeconomic status	−0.042	0.032	−1.297	0.195	[−0.106, 0.022]	
	AIA dimension × SSES	0.107	0.031	3.450	<0.001	[0.046, 0.167]	
	Gender	−0.005	0.056	−0.088	0.930	[−0.114, 0.104]	
	Age	0.000	0.029	−0.003	0.998	[−0.057, 0.057]	
AI-configuration anxiety	Intercept	−0.036	0.084	−0.427	0.670	[−0.200, 0.129]	0.122
	Social media fatigue	0.156	0.039	4.059	<0.001	[0.081, 0.232]	
	AIA dimension	0.175	0.037	4.747	<0.001	[0.102, 0.247]	
	Subjective socioeconomic status	−0.046	0.032	−1.415	0.157	[−0.109, 0.018]	
	AIA dimension × SSES	0.104	0.031	3.337	<0.001	[0.043, 0.165]	
	Gender	0.017	0.055	0.310	0.757	[−0.091, 0.126]	
	Age	0.000	0.029	0.012	0.990	[−0.057, 0.058]	

Note. Continuous variables were standardized. Gender was coded 1 = male and 2 = female. The R^2 value is reported once for each dimension-specific equation. HC3 *SE* = heteroskedasticity-consistent standard error, type 3; CI = confidence interval.

S4. Models without Demographic Covariates

S4.1. Supplementary Methods

Because age and gender were included as covariates in the primary analysis, we examined whether the findings depended on this adjustment. Both equations of the moderated-mediation model were re-estimated without the demographic covariates. The mediator equation included social media fatigue as the sole predictor of AIA, and the outcome equation included social media fatigue, AIA, SSES, and the AIA × SSES product term. Continuous variables remained standardized, HC3 inference was retained, and conditional indirect associations were estimated from 5000 percentile-bootstrap resamples using seed 20260903.

S4.2. Supplementary Results

The results closely reproduced the primary pattern. Social media fatigue remained positively associated with AIA (β = 0.662, HC3 *SE* = 0.037, $p < 0.001$), and the AIA × SSES interaction remained positive and significant in the outcome equation (β = 0.109, HC3 *SE* = 0.031, 95% CI [0.048, 0.170], $p < 0.001$; Table S4). The conditional indirect associations were 0.077 (95% bootstrap CI [0.009, 0.144]) at low SSES, 0.149 (95% bootstrap CI [0.097, 0.205]) at mean SSES, and 0.222 (95% bootstrap CI [0.153, 0.293]) at high SSES. The index of moderated mediation was 0.072 (95% bootstrap CI [0.033, 0.114]). Thus, omitting age and gender did not alter the substantive conclusions.

Table S4. Outcome equation without age and gender.

Predictor	β	HC3 *SE*	*t*	*p*	95% CI
Intercept	−0.015	0.027	−0.547	0.585	[−0.069, 0.039]
Social media fatigue	0.102	0.040	2.584	0.010	[0.025, 0.180]
Artificial intelligence anxiety	0.225	0.041	5.524	<0.001	[0.145, 0.306]
Subjective socioeconomic status	−0.057	0.031	−1.815	0.070	[−0.119, 0.005]
Artificial intelligence anxiety × SSES	0.109	0.031	3.517	<0.001	[0.048, 0.170]

Note. $N = 1108$. Continuous variables were standardized; $R^2 = 0.138$. HC3 *SE* = heteroskedasticity-consistent standard error, type 3; CI = confidence interval.

S5. Distributional Sensitivity for the PHQ-2 Outcome

S5.1. Supplementary Methods

The primary outcome model treated the standardized PHQ-2 sum as a continuous variable. To assess whether the findings were sensitive to this specification, we re-estimated the outcome equation for the original 0–6 PHQ-2 sum using a Poisson model with a log link and the same predictors and covariates. HC3 standard errors were retained, and exponentiated coefficients are reported as incidence-rate ratios (IRRs). We also examined Pearson dispersion and fitted a negative-binomial model as a secondary comparison.

S5.2. Supplementary Results

The Poisson model produced the same substantive pattern as the primary linear model. The AIA × SSES coefficient was positive and statistically significant ($b = 0.070$, HC3 *SE* = 0.018, $p < 0.001$), with an IRR of 1.073 (95% CI [1.035, 1.112]; Table S5). The Pearson dispersion estimate was 0.957, providing no indication of overdispersion. The negative-binomial estimate was numerically equivalent, its estimated dispersion parameter was very large, and its AIC exceeded that of the Poisson model by two points. These results indicate that the focal interaction did not depend on treating the PHQ-2 sum as a continuous outcome.

Table S5. Poisson sensitivity model for the PHQ-2 sum score.

Predictor	*b*	HC3 *SE*	*z*	*p*	IRR	95% CI for IRR
Intercept	0.457	0.070	6.517	<0.001	1.580	[1.377, 1.813]
Social media fatigue	0.075	0.034	2.162	0.031	1.077	[1.007, 1.153]
Artificial intelligence anxiety	0.178	0.034	5.210	<0.001	1.195	[1.117, 1.277]
Subjective socioeconomic status	−0.084	0.027	−3.087	0.002	0.919	[0.871, 0.970]
Artificial intelligence anxiety × SSES	0.070	0.018	3.854	<0.001	1.073	[1.035, 1.112]
Gender	0.017	0.046	0.362	0.717	1.017	[0.929, 1.113]
Age	−0.007	0.024	−0.301	0.763	0.993	[0.948, 1.040]

Note. b is on the log-rate scale; IRR = incidence-rate ratio. Continuous predictors were standardized. Gender was coded 1 = male and 2 = female. HC3 *SE* = heteroskedasticity-consistent standard error, type 3.

S6. Johnson–Neyman Analysis

S6.1. Supplementary Methods

To characterize the interaction beyond the three values used for the simple-slope analysis in the main article, we applied the Johnson–Neyman procedure across the observed range of standardized SSES. At each value of SSES (*W*), the conditional AIA–depressive-symptoms slope was calculated as $b_1 + b_3W$. Its standard error incorporated the HC3 variances of the AIA and interaction coefficients and their covariance. Region boundaries were identified where the two-sided 95% confidence interval touched zero, using the residual degrees of freedom from the outcome equation.

S6.2. Supplementary Results

Standardized SSES ranged from −2.464 to 2.480 in the observed sample. The two mathematical roots were −4.900 and −1.099 *SD*, of which only −1.099 fell within the observed range. Using the raw-score mean of 5.485 and *SD* of 1.820, the within-range root corresponds to a raw SSES score of 3.484 on the 1–10 scale. Because the two-item mean occurred in half-point increments, 120 participants (10.8%) with observed scores of 3.0 or lower fell below this boundary, whereas the conditional AIA–depressive-symptom association was positive and statistically distinguishable from zero at observed scores of 3.5 or higher. This is a sample-specific region-of-significance boundary, not a substantive socioeconomic cut-off.

S6.3. Interpretation in the Original PHQ-2 Metric

The primary outcome model used standardized AIA and standardized PHQ-2 scores. To express the conditional association in the original 0–6 PHQ-2 metric, each standardized simple slope was multiplied by the observed PHQ-2 standard deviation (1.38). A two-standard-deviation AIA contrast was calculated by doubling the corresponding one-standard-deviation contrast. These quantities are adjusted model-implied contrasts; they are not observed differences between participant groups and should not be interpreted as clinical change.

Table S6. Conditional AIA associations and model-implied contrasts in original PHQ-2 units.

SSES level	Standardized SSES	Raw SSES value	Standardized AIA slope [95% CI]	PHQ-2 points per 1-*SD* higher AIA [95% CI]	PHQ-2 difference: AIA −1 *SD* to +1 *SD* [95% CI]
Low	−1	3.66	0.116 [0.015, 0.218]	0.16 [0.02, 0.30]	0.32 [0.04, 0.60]

SSES level	Standardized SSES	Raw SSES value	Standardized AIA slope [95% CI]	PHQ-2 points per 1-*SD* higher AIA [95% CI]	PHQ-2 difference: AIA −1 *SD* to +1 *SD* [95% CI]
Mean	0	5.48	0.226 [0.146, 0.306]	0.31 [0.20, 0.42]	0.62 [0.40, 0.84]
High	+1	7.31	0.335 [0.236, 0.435]	0.46 [0.33, 0.60]	0.92 [0.65, 1.20]

Note. N = 1108. PHQ-2 *SD* = 1.38. Continuous predictors and the primary outcome were standardized for model estimation. Values are rounded; calculations used unrounded coefficients. AIA = artificial intelligence anxiety; SSES = subjective socioeconomic status; CI = confidence interval.

S7. Software and Reproducibility

All primary and supplementary analyses were conducted in R (v4.4.1). The principal packages were psych (v2.5.3), sandwich (v3.1-1), lmtest (v0.9-40), car (v3.1-3), MASS (v7.3-60.2), and ggplot2 (v4.0.2). Confirmatory factor analyses were estimated in Mplus (v8.3) with MLR. The primary conditional indirect associations were based on 5000 percentile-bootstrap resamples using seed 20260902; the corresponding no-covariate analysis used seed 20260903.

S8. Social Media Fatigue Items and Measurement Evidence

Table S8 reproduces the exact Chinese-language items administered in the survey and literal English translations. Responses ranged from 1 (strongly disagree) to 5 (strongly agree). A one-factor confirmatory factor analysis was estimated with robust maximum likelihood. All five items loaded strongly on the intended factor, but the fit indices provide mixed evidence because RMSEA was elevated and the inter-item correlations were high. The complete five-item scale was retained to preserve the content and scoring of the published Chinese measure.

Table S8. Social media fatigue items and one-factor measurement model.

Item	Chinese wording administered	Literal English translation	Standardized loading
SMF1	持续使用社交媒体后，我很难放松下来。	After continuously using social media, I find it difficult to relax.	0.889
SMF2	使用一段时间社交媒体后，我真的感觉很疲惫。	After using social media for a period of time, I really feel fatigued.	0.894
SMF3	由于使用社交媒体，我感觉相当疲惫。	Because of using social media, I feel rather drained.	0.906
SMF4	使用社交媒体后，我在闲暇时间很难集中注意力。	After using social media, I find it difficult to concentrate during my leisure time.	0.853
SMF5	在使用社交媒体时，我经常感觉太累，无法做好其他事情。	When using social media, I often feel too tired to do other things well.	0.845

Note. Robust maximum likelihood (MLR). Scaled $\chi^2(5)$ = 36.77, robust CFI = 0.974, robust TLI = 0.948, robust RMSEA = 0.155, and SRMR = 0.025. Standardized loadings ranged from 0.845 to 0.906; inter-item correlations ranged from 0.721 to 0.826; Cronbach's α = 0.944. RMSEA can be inflated in models with few degrees of freedom, but the elevated value is retained and may also reflect localized misfit or closely overlapping item content.

S9. Distribution of PHQ-2 Sum Scores

The PHQ-2 sum ranged from 0 to 6. Table S9 reports the complete observed distribution. Scores of 3 or higher are shown descriptively as a conventional screening threshold; they are not treated as a diagnosis of depressive disorder in this study.

Table S9. Distribution of PHQ-2 sum scores.

PHQ-2 score	*n*	%
0	295	26.6
1	126	11.4
2	482	43.5
3	84	7.6
4	93	8.4
5	1	0.1
6	27	2.4
≥3 (screening threshold)	205	18.5

Note. N = 1108. Mean = 1.70, *SD* = 1.38, skewness = 0.70, and excess kurtosis = 0.68. The Poisson dispersion statistic was 0.96 (Table S5), indicating no overdispersion.

S10. Subjective Socioeconomic Status Items and Item-Specific Analyses

The two SSES items used different reference groups but both assessed perceived relative position. Panel A reproduces the exact Chinese wording, literal English translations, and response anchors. Panel B reports the moderation analyses using each item separately and the two-item mean used in the primary model. The item-specific models are sensitivity analyses, not a complete construct-validation study.

Table S10. Exact SSES item wording and item-specific moderation analyses.

Panel A. Exact item wording

Item	Chinese wording administered	Literal English translation	Response anchors
SSES1	请根据您所在省份的社会经济发展状况，确定您家庭的经济地位	Based on the socioeconomic development of the province where you live, please indicate your family's economic position.	1 = lowest; 10 = highest
SSES2	请与您周围的人群相比，确定您的社会经济地位	Compared with people around you, please indicate your socioeconomic position.	1 = lowest; 10 = highest

Panel B. Item-specific moderation estimates

Moderator	Interaction β [95% CI]	ΔR^2	Slope at −1 *SD* [95% CI]	Slope at +1 *SD* [95% CI]	Index of moderated mediation [95% CI]
Item 1: family position within province	0.097 [0.036, 0.158]	0.016	0.133 [0.032, 0.233]	0.327 [0.225, 0.429]	0.064 [0.025, 0.105]
Item 2: own position relative to people nearby	0.115 [0.055, 0.175]	0.025	0.110 [0.009, 0.210]	0.340 [0.240, 0.439]	0.076 [0.038, 0.117]
Two-item mean (primary model)	0.110 [0.049, 0.171]	0.022	0.116 [0.015, 0.218]	0.335 [0.236, 0.435]	0.072 [0.034, 0.113]
Both item interactions entered together	Item 1: −0.040 [−0.125, 0.044]; Item 2: 0.150 [0.065, 0.235]	—	—	—	—

Note. N = 1108. The two items correlated *r* = 0.86. Continuous variables were standardized separately for each model. All models controlled for social media fatigue, age, and gender and used HC3 robust standard errors. Indices of moderated mediation used 5000 percentile-bootstrap resamples (seed 20260902). CI = confidence interval; SSES = subjective socioeconomic status.

S11. Regression Diagnostics and Additional Sensitivity Analyses

Regression diagnostics assessed multicollinearity, heteroscedasticity, studentized residuals, leverage, and Cook's distance for both primary equations. Maximum VIF values were low, but Breusch–Pagan tests indicated heteroscedasticity; HC3 robust standard errors were therefore used for inference throughout. No observation had Cook's D greater than 1. Counts based on conventional screening rules were treated as diagnostic flags rather than automatic exclusion criteria.

Table S11. Regression diagnostics and additional sensitivity analyses.

Panel A. Primary-model diagnostics

Equation	Maximum VIF	Breusch–Pagan *p*	Maximum Cook's *D*	*n* with Cook's *D* > 4/*N*	*n* with ∣studentized residual∣ > 3	Maximum leverage
Mediator: AIA	1.02	<0.001	0.084	109	18	0.019
Outcome: PHQ-2	1.82	<0.001	0.041	80	14	0.060

Note. N = 1108. VIF = variance inflation factor. The Cook's-distance screening threshold was 4/*N*. The diagnostic flags were not prespecified exclusion rules; HC3 inference and an outcome-model influence sensitivity analysis were used instead.

Panel B. Influence, response-pattern, and gender sensitivity analyses

Analysis	*n*	*r* (SMF, AIA)	AIA × SSES β [95% CI]	ΔR^2	Indirect at −1 *SD* [95% CI]	Indirect at +1 *SD* [95% CI]	Index [95% CI]
Primary model	1108	0.662	0.110 [0.049, 0.171]	0.022	0.077 [0.010, 0.144]	0.221 [0.156, 0.292]	0.072 [0.034, 0.113]
Excluding outcome-model Cook's D > 4/N	1028	—	0.121 [0.074, 0.169]	—	—	—	—
Excluding identical responses across all 26 SMF and AIA items	624	0.455	0.104 [0.004, 0.205]	0.013	0.073 [0.011, 0.133]	0.166 [0.107, 0.236]	0.047 [0.006, 0.093]
Excluding identical responses across all 21 AIA items	507	0.541	0.150 [0.027, 0.272]	0.027	0.058 [−0.022, 0.146]	0.219 [0.135, 0.310]	0.080 [0.018, 0.142]
Men only	690	—	0.101 [0.029, 0.172]	—	—	—	—
Women only	418	—	0.119 [0.019, 0.219]	—	—	—	—
AIA × SSES × gender	1108	—	0.025, *p* = 0.695	—	—	—	—

Note. AIA = artificial intelligence anxiety; SMF = social media fatigue; SSES = subjective socioeconomic status; CI = confidence interval. Identical responding was examined as a sensitivity condition, not as proof of invalid responding. Of the 484 participants giving the same response to all 26 SMF and AIA items, 306 (63.2%) varied on the PHQ-2 or SSES items or reported SSES outside the middle range (mean ≤ 4 or ≥ 7). Response time was not available in the analytic dataset, and no response-time exclusion threshold

was prespecified. Men reported slightly higher AIA than women ($M = 3.08$ vs. 2.95; Welch $t = 3.45$, $p < 0.001$). Bootstrap intervals are based on 5000 resamples. Em dashes indicate quantities that were not estimated for that sensitivity model or were not applicable.
